# A Systematic Review of Fecal Microbiota Transplant for the Management of Pouchitis

**DOI:** 10.1093/crocol/otaa034

**Published:** 2020-05-12

**Authors:** Maia Kayal, Thomas Lambin, Rachel Pinotti, Marla C Dubinsky, Ari Grinspan

**Affiliations:** 1 Department of Medicine, Division of Gastroenterology, Icahn School of Medicine at Mount Sinai, New York, New York, USA; 2 Division of Gastroenterology, Lille University Hospital, Lille, France; 3 Library Education & Research Services, Icahn School of Medicine at Mount Sinai, New York, New York, USA

**Keywords:** pouchitis, fecal microbiota transplant

## Abstract

**Background:**

Manipulation of the pouch microbiota via fecal microbiota transplant (FMT) has been theorized to be a promising therapeutic approach for pouchitis. The goal of this systematic review was to summarize the available, high-quality data on the efficacy and safety of FMT for acute and chronic pouchitis.

**Methods:**

A systematic electronic literature search was conducted on Embase, MEDLINE, Scopus, and Cochrane CENTRAL. Randomized controlled trials and observational studies that assessed the efficacy and safety of FMT for the treatment of acute and/or chronic pouchitis in patients with ulcerative colitis who underwent total proctocolectomy with ileal pouch-anal anastomosis were included.

**Results:**

Four studies involving the use of FMT for chronic pouchitis were considered eligible for data extraction. No study involving the use of FMT for the management of acute pouchitis was identified. In 1 study, 3/5 (75%) patients achieved sustained clinical remission at 3 months. In the remaining 3 studies, 2/8, 1/11, and 1/5 patients achieved clinical response defined as a decrease in pouchitis disease activity index at least 3. Stool donor engraftment as determined by 16s rRNA gene sequencing occurred only in those patients with clinical response.

**Conclusions:**

The 4 studies that met inclusion criteria for this systematic review indicate FMT is safe in chronic pouchitis, however largely not efficacious. These data are limited by study heterogeneity. Additional studies are required to guide the use of FMT in patients with acute and chronic pouchitis.

## INTRODUCTION

The staged total proctocolectomy (TPC) with ileal pouch-anal anastomosis (IPAA) is the gold standard surgical treatment for ulcerative colitis (UC) complicated by medically refractory disease or dysplasia. Pouchitis is the most common post-IPAA inflammatory condition with reported cumulative incidence rates of 25% at 1 year, 35% at 3 years, and 45% at 5 years.^1^ Antibiotics are the mainstay of treatment and induce remission rates of 80%; however, approximately 20% of patients progress to chronic pouchitis with an associated 5%–10% risk of pouch failure.^2–4^

Intestinal dysbiosis is widely believed to occur after TPC with IPAA and plays a role in the development of pouchitis. The construction of the ileal pouch leads to altered bowel anatomy that promotes colonic metaplasia in the pouch mucosa, creating a molecular environment favorable to the development of inflammation in the context of fecal stasis.^5^ This theory is supported by the clinical observation that pouch inflammation does not occur before the restoration of intestinal continuity and exposure of the pouch to the fecal stream.^6^ The particular microbiota that trigger pouch inflammation is unknown; however, Enterobacteriaceae, Fusobacterium, and *Clostridia* spp. in the context of decreased bacterial diversity have been implicated.^5, 7–13^

Given the significant role dysbiosis plays in the pathogenesis of pouch inflammation, manipulation of the pouch microbiota via fecal microbiota transplant (FMT) has been theorized to be a promising therapeutic approach for pouchitis. Unfortunately, multiple small case series and case studies have reported conflicting results regarding the efficacy of FMT in the management of acute and chronic pouchitis. This is due to significant heterogeneity in analysis techniques, sampling strategies, route of administration, and frequency of transplant. The goal of this systematic review was to summarize the available, high-quality data on the efficacy and safety of FMT for acute and chronic pouchitis.

## METHODS

### Search Strategy and Eligibility Criteria

A comprehensive search comprised of both index terms and keywords was executed in the Embase, MEDLINE, Scopus, and Cochrane CENTRAL databases. No date, language, or other search filters were utilized. The full-electronic search strategy for all databases is reported in Supplementary Appendix A.

Randomized controlled trials and observational studies that assessed the efficacy and safety of FMT for the treatment of acute and/or chronic pouchitis in patients with UC who underwent TPC with IPAA were included. Retrospective case series and case studies were excluded. Studies of all patients, both adult and pediatric, with evidence of active clinical and endoscopic pouchitis (acute, chronic antibiotic refractory, or chronic antibiotic dependent) were eligible.

### Study Selection and Data Extraction

**Figure 1.**
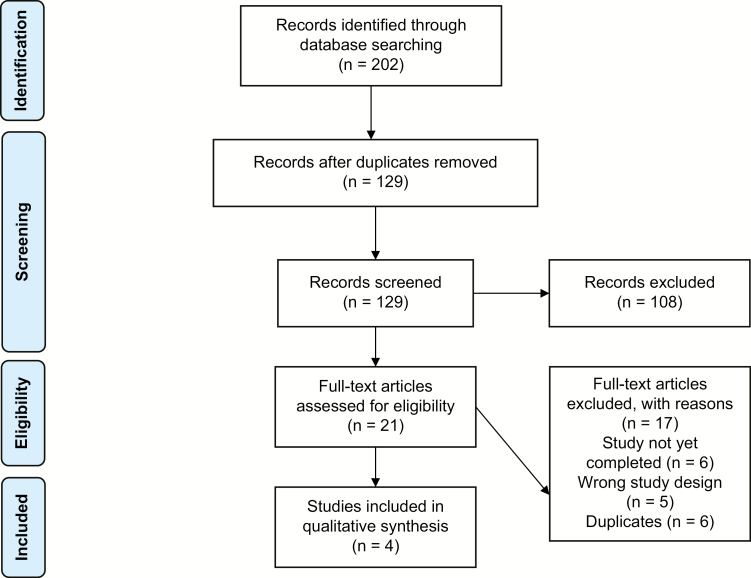
PRISMA flow diagram.

All search results were imported into the Covidence online systematic review software screened according to predefined eligibility criteria. Initial screening was conducted on the basis of article titles and abstracts. Results that met the eligibility or could not be conclusively excluded were screened on the basis of the full text. Both rounds of screening were conducted independently by 2 reviewers (M.K. and T.L.), and any discrepancy was settled by a third reviewer (A.G.). The screening results are shown in Figure 1.

Eligible articles were reviewed by M.K. and T.L. Results from the included articles were extracted into tables. The proportions of patients who achieved clinical response, clinical remission, endoscopic response, and/or donor engraftment as defined by each study were derived.

## RESULTS

The literature search identified a total of 202 studies. After removing duplicates, 129 studies remained for the initial title and abstract screening. After screening, 19 studies remained for full-text assessment. After full-text review, 15 studies were excluded for wrong study design, ongoing recruitment, or abstract duplication. A total of 4 studies were considered eligible for data extraction (Table 1). All identified studies involved the use of FMT for the management of chronic pouchitis. No study involving the use of FMT for the management of acute pouchitis was identified.

**Table 1.** Included Studies on FMT for Chronic Pouchitis

Author	Year	Study Design	Sample Size	Donor Source	Route of FMT	Frequency	Antibiotic Use	Probiotic Use	Clinical Response*	Clinical Remission*	Endoscopic Response*	Donor Engraftment
Landy et al	2015	Cohort	8	Multiple, related, and unrelated	Nasogastric infusion	1	No antibiotics for 2 weeks before	Not discussed	2/8	0/8	Not assessed	0/8
Stallmach et al	2016	Cohort	5	2 donors, unrelated	Endoscopy	1–7	No antibiotics permitted at the time of study initiation	No probiotics permitted at the time of study initiation	5/5	3/5	5/5	2/5
Selvig et al	2019	Clinical trial	19	13 donors, unrelated (stool bank)	Pouchoscopy	1–2	Rifaximin pre-FMT in 8 patients	Probiotics in 9 patients at the initiation of study	1/11	0/19	0/19	2/19
Herfarth et al	2019	RCT	6	1 donor, unrelated (stool bank)	Pouchoscopy, oral capsules	2 pouchoscopy, 14 days oral capsules	Ciprofloxacin, metronidazole, amoxicillin in all patients stopped 24 hours pre-FMT	Not discussed	1/5	1/6	Not assessed	1/6

*Outcomes were defined differently in each study.

Landy et al^14^ conducted a pilot study of FMT in 8 patients with chronic antibiotic refractory pouchitis “diagnosed clinically, endoscopically, and histologically with a current PDAI ≥7.” Stool samples were derived from healthy relatives and partners or anonymous unrelated donors. Antibiotics were discontinued for 2 weeks before study initiation; probiotics were not discussed in the context of the study protocol. Baseline pouchoscopy was performed, FMT was delivered via nasogastric infusion, and repeat pouchoscopy was performed 4 weeks later. All patients received a single FMT. Pre- and post-FMT pouch inflammation was assessed via the composite Pouchitis Disease Activity Index (PDAI) score. No patient achieved clinical remission. Two patients had a reduction of composite PDAI score at least 3 at 4 weeks post-FMT; however, both still had a composite PDAI score at least 7. No major adverse events following FMT were observed. There were no overall changes in bacterial diversity after FMT; however, nonmetric multidimensional scaling revealed a shift in the stool microbiome of the 2 patients who had a reduction in PDAI toward a composition similar to that of the stool donor.

Stallmach et al^15^ conducted an observational study of FMT in 5 patients with chronic antibiotic refractory pouchitis. The definition of chronic antibiotic refractory pouchitis was not provided. Stool samples were derived from 2 unrelated healthy donors. Antibiotic or probiotic use was not permitted at the time of study initiation. Baseline pouchoscopy was performed, FMT was delivered via direct administration into the jejunum during esophagogastroduodenoscopy, and repeat pouchoscopy was performed within 3 months. Pre- and post-FMT pouch inflammation was assessed via composite PDAI scores in all patients and calprotectin in a subset of 3 patients. Follow-up was recorded for 3 months. Four of the 5 patients in the study required 3 or more FMT administrations. Symptoms resolved in 4 patients, PDAI scores improved in all 5 patients, and calprotectin values decreased in the 3 patients with available data within 4 weeks of their last FMT. Sustained response at 3 months after the last FMT was observed in 3 patients and no further antibiotic courses were required. One patient developed a mild transient fever after FMT, and no major adverse events were otherwise noted. Deep sequencing on the genus level (V1V2 region of the 16S rRNA gene) revealed distinct microbial community changes after FMT in the 3 patients who received the same donor stool. In the 2 patients who responded of the 3, the stool microbiome reflected that of the donor.

Selvig et al^16^ conducted a prospective, open-label pilot study of FMT in 19 patients with chronic pouchitis, defined as endoscopic evidence of pouch inflammation with increased stool frequency, urgency, tenesmus, or hematochezia lasting greater than 4 weeks. Stool samples were obtained from a universal stool bank from 13 donors, with each FMT procedure containing nonpooled donor stool from 1 individual. Seven patients received rifaximin before FMT to facilitate engraftment, and 9 patients were on probiotics at the time of study initiation. Baseline pouchoscopy was performed, FMT was delivered via pouchoscopy into the proximal pouch or neo-terminal ileum, and repeat pouchoscopy was performed 4 weeks later. The primary outcome was clinical improvement in pouchitis at 4 weeks after FMT assessed by patient surveys; secondary outcomes included decreases in composite PDAI at least 3 at week 4, erythrocyte sedimentation rate (ESR), C-reactive protein (CRP), and fecal calprotectin compared to baseline. Eighteen patients completed follow-up at 4 weeks. Eleven patients electively underwent a repeat FMT at 4 weeks. There was a statistically significant improvement in bowel movement frequency from 9.25 movements per day pre-FMT to 7.25 post-FMT, *P* = 0.03. PDAI was available for 11 patients, and decreased from a median of 7 pre-FMT to 6 four weeks post-FMT (*P* = 0.33), with only 1/11 patients achieving the secondary outcome of PDAI reduction at least 3. ESR and CRP values did not decrease significantly after FMT. Calprotectin values were available for 4 patients pre- and post-FMT and demonstrated a decrease from median 344 to 200. Four patients required an additional course of antibiotics within 4 weeks of FMT, and 3 patients required escalation to biologic therapy within 12 months. No deaths, infectious complications, or related hospitalizations were observed at 4 weeks after FMT, though 2 patients developed Crohn’s-like disease of the pouch. Microbiome sequencing (V4 region of the 16S rRNA gene) revealed no distinct community-level changes after FMT. In the single patient who had an improvement of PDAI greater than 3 points, the stool microbiome after FMT reflected that of the donor.

Herfarth et al^17^ conducted a prospective, placebo-controlled double-blind trial of endoscopic and oral FMT in patients with chronic antibiotic-dependent pouchitis, defined as (1) the need for greater than 4 weeks of antibiotic therapy to maintain clinical remission and at least 2 episodes of pouchitis following antibiotic discontinuation in the last 24 months, or (2) active pouchitis with a modified PDAI at least 5 and a history of at least 4 antibiotic courses in the preceding 12 months. Stool samples were derived from a single stool donor from a stool bank. All patients received antibiotic therapy (ciprofloxacin, metronidazole, or amoxicillin) to induce clinical remission, discontinued at least 24 hours before FMT. Probiotic use was not discussed in the context of the study protocol. Patients were randomized to undergo active endoscopic or placebo FMT followed by daily active oral FMT for 14 days. FMT was delivered via pouchoscopy. Patients who experienced a relapse within the first 4 weeks after FMT were offered repeat endoscopic FMT followed by daily oral FMT for 2 weeks. The primary outcome was the safety of combined endoscopic and oral FMT; secondary outcomes included modified PDAI less than 4 with no need for antibiotics in weeks 4, 8, and 16 after endoscopic FMT, quantitative changes in calprotectin, and engraftment of donor FMT. Four patients were randomized to active FMT and 2 patients to inactive placebo FMT. All patients had an increase in diarrhea, urgency, and calprotectin during or shortly after FMT. After re-induction with antibiotics, 5 patients proceeded with the open-label active arm and repeat endoscopic FMT followed by daily oral FMT for 2 weeks. One achieved clinical remission and 4 had an increase in clinical symptoms during or shortly after FMT. No FMT-related safety events occurred. Recruitment was halted due to the low efficacy of FMT. Microbiome sequencing (V4 region of the 16S rRNA gene) revealed donor engraftment only in the 1 patient who achieved clinical remission.

## DISCUSSION

The 4 studies that met inclusion criteria for this systematic review indicate FMT is safe in chronic pouchitis, however largely not efficacious. Clinical remission and response as defined by a decrease in composite PDAI score at least 3 were reported in only a few patients, and donor engraftment was observed exclusively in these select few. Given the relatively few included studies, however, these results should be interpreted with caution. Drawing aggregate conclusions from these data is difficult since overall sample sizes were small, inclusion criteria were different, and outcomes were not standardized.

The ability to manipulate the microbiome through FMT has proven to be effective in patients with UC. In a comprehensive systematic review and meta-analysis assessing the effectiveness and safety of FMT in inflammatory bowel disease, significant benefit in clinical remission (pooled odds ratio = 2.89, 95% CI = 1.36–6.13, *P* = 0.006) with moderate heterogeneity was noted in patients with UC.^18^ Sub-analyses reported further improved remission rates with an increased number of FMT infusions and lower gastrointestinal tract administration. FMT would be expected to yield at least similar rates of clinical remission in chronic pouchitis to those observed in UC given the central role intestinal dysbiosis plays in its pathogenesis. However, small case series and case studies have revealed mixed results, with the majority showing no efficacy of FMT in chronic pouchitis.^19, 20^ Although the reasons for these results are not entirely clear, one potential consideration is the source of donor stool. All studies evaluating the efficacy of FMT in chronic pouchitis to date have used stool from healthy donors with full colons, rather than stool from healthy donors who have undergone TPC with IPAA. Although colonic metaplasia occurs in the pouch after the restoration of intestinal continuity, the microbiome of the healthy pouch remains inherently different than that of the healthy colon. A more appropriate transplant protocol could be to use stool from healthy patients who have undergone TPC with IPAA for familial adenomatous polyposis (FAP). Pouchitis is less common in patients with FAP compared to patients with UC, with cumulative incidence rates of 0%–20%, thus suggesting they harbor a healthier baseline microbiome.^2, 21–23^ This healthier pouch microbiome may be the key to proving FMT effective in the management of chronic pouchitis.

The data included in this systematic review are difficult to interpret in congregate given the significant heterogeneity of the underlying studies. First, chronic pouchitis and treatment outcomes such as clinical response, clinical remission, and endoscopic response were defined differently in each study due to the lack of published criteria for chronic inflammatory pouch conditions. This made it difficult to standardize participants and efficacy results across the studies. Second, stool samples were derived from different sources in each study (related and unrelated healthy donors, universal stool bank), and only 1 study provided information on whether samples were pooled. Third, stool samples were delivered via different routes (esophagogastroduodenoscopy, nasogastric tube, pouchoscopy) and were therefore deposited in different anatomic locations. The delivery route has been shown to impact the efficacy rates of FMT in the treatment of recurrent *Clostridioides difficile*, with delivery via colonoscopy producing greater efficacy rates.^24^ Similar differences in efficacy could be observed in the setting of pouchitis, particularly when considering the significant fecal stasis and localized dysbiosis that occurs in the pouch body compared to the pre-pouch inflow tract. Fourth, the frequency of FMTs differed in each study, with 1 study permitting up to 7. Fifth, and perhaps most importantly, antibiotic and probiotic use at the time of FMT was unique in each study. Two studies used antibiotics to induce clinical remission before FMT, 1 study mandated antibiotic discontinuation 2 weeks before FMT, and 1 study mandated antibiotic discontinuation only 24 hours before FMT. Similarly, clarity regarding probiotic use was lacking. Probiotic use at the time of study initiation was permitted in 1 study, prohibited in another study, and not discussed in the remaining 2 studies. Antibiotics and probiotics each have the potential to significantly manipulate the microbiome.^25^ Different doses and durations of antibiotics and probiotics make the interpretation and analysis of the microbiome in the context of FMT difficult.

Additional studies are needed to provide clarity on the efficacy of FMT for pouchitis, specifically utilizing standardized definitions for inflammatory pouch conditions and outcomes, and addressing important considerations such as the use of fresh or frozen stool from pooled or nonpooled multi-donors, frequency and interval of FMT, and concomitant use of antibiotics and probiotics. There are 3 ongoing studies registered on ClinicalTrials.gov involving the use of FMT for pouchitis that have the potential to shed light on these important considerations.

## CONCLUSION

In summary, there is inadequate data to recommend FMT for chronic pouchitis at this time. Until the results from the aforementioned studies are available, the use of FMT for chronic pouch inflammatory conditions should be done on an individual basis.

## DATA AVAILABILITY

The studies included in this systematic review were obtained from the Embase, MEDLINE, Scopus, or Cochrane CENTRAL databases. The digital object identifiers for the included studies are 10.1038/srep12955, 10.1038/ajg.2015.436, 10.1007/s10620-019-05715-2, and 10.1159/000497042.

## Supplementary Material

otaa034_suppl_Supplementary_Appendix_A

## Notes

Authorship: Guarantor of the article: M.K. is the submission’s guarantor and takes responsibility for the integrity of the work as a whole, from inception to published article.

Author contributions: M.K.: study concept and design, acquisition of data, analysis and interpretation of data, drafting of the manuscript, critical revision of the manuscript for important intellectual content, and statistical analysis; T.L.: study concept and design, acquisition of data, and critical revision of the manuscript for important intellectual content; R.P.: study concept and design, search strategy execution, and acquisition and management of data; M.C.D.: study concept and design and critical revision of the manuscript for important intellectual content; A.G.: study concept and design and critical revision of the manuscript for important intellectual content. All authors approved the final version of the manuscript.
