# Supplementary material for: A Systematic Review of Fecal Microbiota Transplant for the Management of Pouchitis
Source: Crohns Colitis 360. 2020 May 12;2(2):otaa034. doi: 10.1093/crocol/otaa034 (PMC9802227; doi:10.1093/crocol/otaa034)

**Appendix A - Full Electronic Search Strategies for All Databases**

# MEDLINE (Ovid)

1. fecal microbiota transplantation/

2. (f?ecal adj2 microbio$ adj2 transplant$).tw.

3. ((f?ecal or stool or intestinal) adj2 (transplant$ or bacteriotherapy)).tw.

4. 1 or 2 or 3

5. pouchitis/

6. (pouchitis or ileal pouch$).tw.

7. 5 or 6

8. 4 and 7

# Embase (Ovid)

1. fecal microbiota transplantation/

2. (f?ecal adj2 microbio$ adj2 transplant$).tw.

3. ((f?ecal or stool or intestinal) adj2 (transplant$ or bacteriotherapy)).tw.

4. 1 or 2 or 3

5. ileitis/

6. (pouchitis or ileal pouch$).tw.

7. 5 or 6

8. 4 and 7

# Scopus

( ( TITLE-ABS-KEY ( ( fecal AND microbiota AND transplantation ) ) OR TITLE-ABS-KEY ( ( fecal OR stool OR intestinal ) W/3 ( transplant$ OR bacteriotherapy ) ) ) ) AND ( TITLE-ABS-KEY ( pouchitis OR "ileal pouch" OR "ileal pouches" ) )

# Cochrane Central


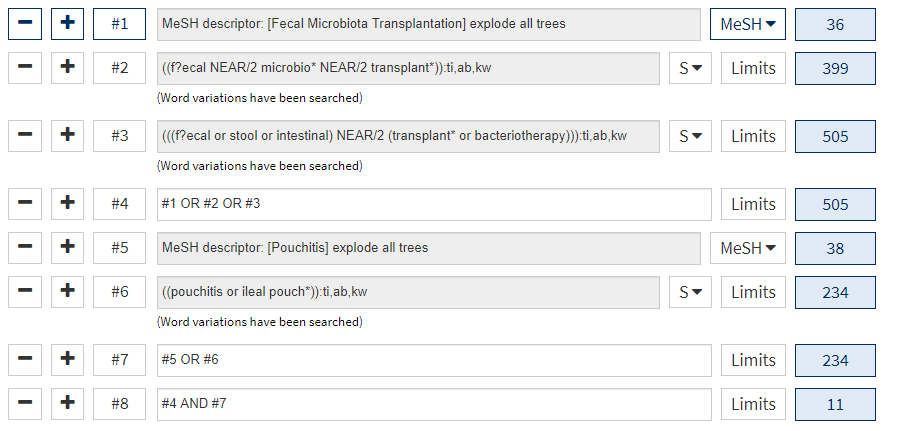

Supplement: otaa034_suppl_Supplementary_Appendix_A [file otaa034_suppl_supplementary_appendix_a.docx]
